# Early‐Onset Delayed Intraparenchymal Hemorrhage Following Flow Diversion: A Case Report Challenging the Windkessel Hypothesis

**DOI:** 10.1002/ccr3.73295

**Published:** 2026-08-10

**Authors:** Feyz Ebrahimnia, Farid Kazemi, Mohammad Hossein Mirbolouk, Sajjad Khanbabazadeh, Mohammad Samadian, Seyad Tahar Mosavyan, Mohammadreza Moznebiisfahani

**Affiliations:** ^1^ Department of Neurosurgery, School of Medicine Iran University of Medical Sciences Tehran Iran; ^2^ Department of Neurosurgery, Skullbase Research Center, Loghman Hakim Medical Center Shahid Beheshti University of Medical Sciences Tehran Iran; ^3^ Department of Neurosurgery, Al‐Zahra Hospital Isfahan University of Medical Sciences Isfahan Iran

**Keywords:** carotid artery injury, flow diverter, pseudoaneurysm, Windkessel effect

## Abstract

Intraoperatively, in a patient with a history of macroadenoma, the cavernous segment of the internal carotid artery was injured. CTA revealed a dissecting pseudoaneurysm at the carotid bifurcation. This case illustrates a fulminant form of DIPH occurring within days of FD deployment.

## Key Clinical Message

Early‐onset delayed intraparenchymal hemorrhage following flow diversion is a rare condition, which should be considered in clinical practice.

## 1 Introduction

Flow diverter stents have emerged as a transformative modality in the treatment of intracranial aneurysms, particularly complex and wide‐necked configurations. Their mechanism of action involves endoluminal reconstruction of the parent artery, redirecting blood flow away from the aneurysm sac and promoting thrombosis and subsequent remodeling [1, 2]. Despite the growing popularity and effectiveness of this approach, rare but devastating complications such as delayed intraparenchymal hemorrhage (DIPH) and delayed aneurysm rupture have increasingly been recognized [3, 4, 5].

One of the primary theories proposed to explain DIPH is the Windkessel hypothesis. This hypothesis posits that the aneurysmal sac provides a degree of compliance in the cerebral circulation that is lost after flow diverter placement, thereby resulting in increased pulse pressure distally, which may predispose to hemorrhagic events [6]. However, the precise role of this mechanism remains controversial.

In this report, we present a unique case of exceptionally early‐onset DIPH and dissecting pseudoaneurysm formation following flow diversion in a patient with prior carotid artery manipulation. The case challenges the classical understanding of post‐FD hemorrhagic complications and supports the growing recognition that these events may be multifactorial.

## 2 Case History

A 46‐year‐old woman with a notable medical history of pituitary macroadenoma, which had been previously treated via transsphenoidal surgery 3 years prior, presented with progressive visual decline. The visual deterioration prompted further investigation, leading to the suspicion of tumor recurrence. Imaging studies indicated changes consistent with possible regrowth of the pituitary mass, necessitating a repeat surgical intervention.

During the surgical procedure aimed at resecting the suspected recurrent tumor, an inadvertent injury occurred to the cavernous segment of the left internal carotid artery (ICA). This injury resulted in significant hemorrhage, which was promptly controlled through surgical packing techniques. The management of intraoperative hemorrhage is critical, especially in neurosurgical settings, where vascular structures are closely associated with the operative field.

## 3 Differential Diagnosis, Investigations, and Treatment

Postoperative digital subtraction angiography (DSA) revealed the presence of a pseudoaneurysm at the cavernous segment of the ICA. Given the potential for serious complications associated with a pseudoaneurysm, the patient was scheduled for an endovascular intervention. A flow diverter stent (Silk Vista 4 × 30, Balt) was successfully deployed to treat the pseudoaneurysm. Initial DSA after stent placement showed no evidence of wall irregularities or compromise beyond the pseudoaneurysm, indicating that the stent was functioning effectively.

Postoperatively, the patient was closely monitored in the intensive care unit (ICU). For the first 72 h, she remained neurologically intact, maintaining a Glasgow Coma Scale (GCS) score of 15 and preserving her visual function. The patient received aspirin 325 mg and ticagrelor 180 mg before the procedure, followed by aspirin 80 mg daily and ticagrelor 90 mg twice daily.

**FIGURE 1:**
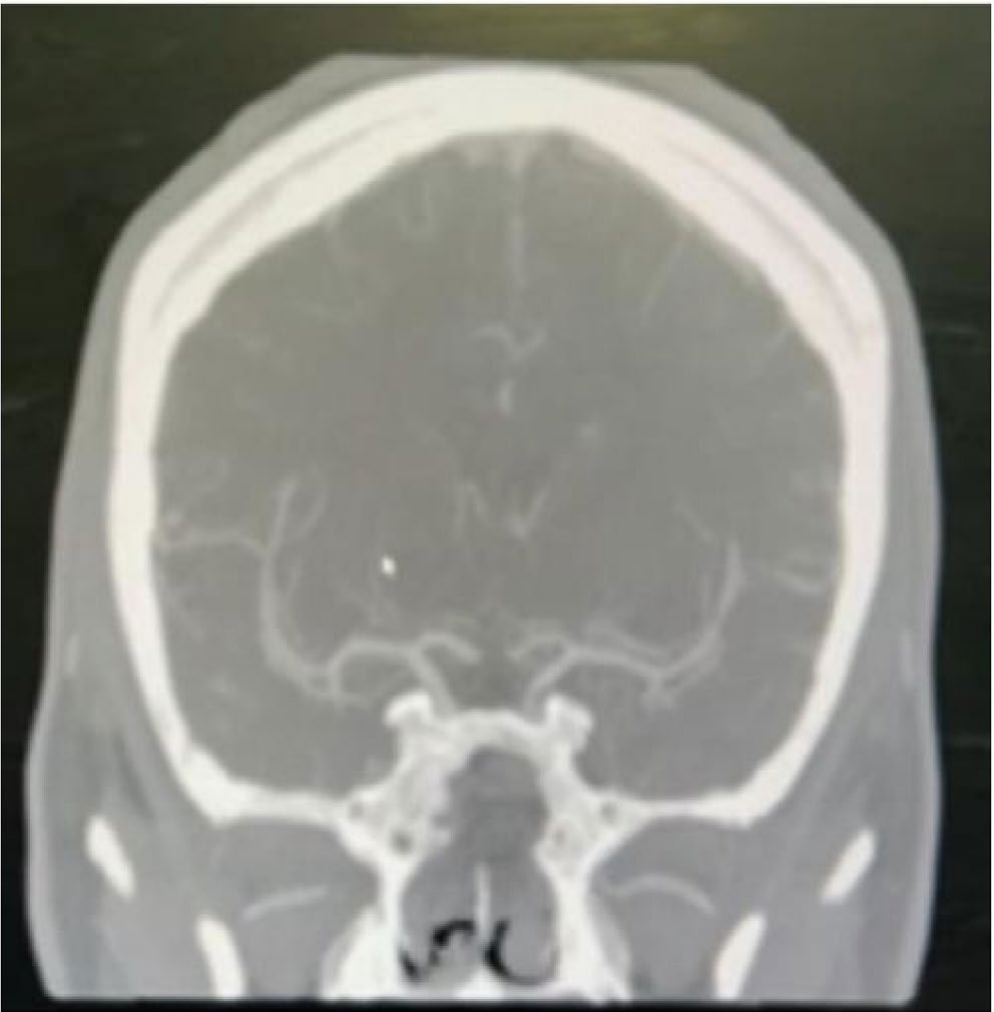
Patient's brain CT angiography that showed a patent stent with no signs of new aneurysm formation.

**FIGURE 2:**
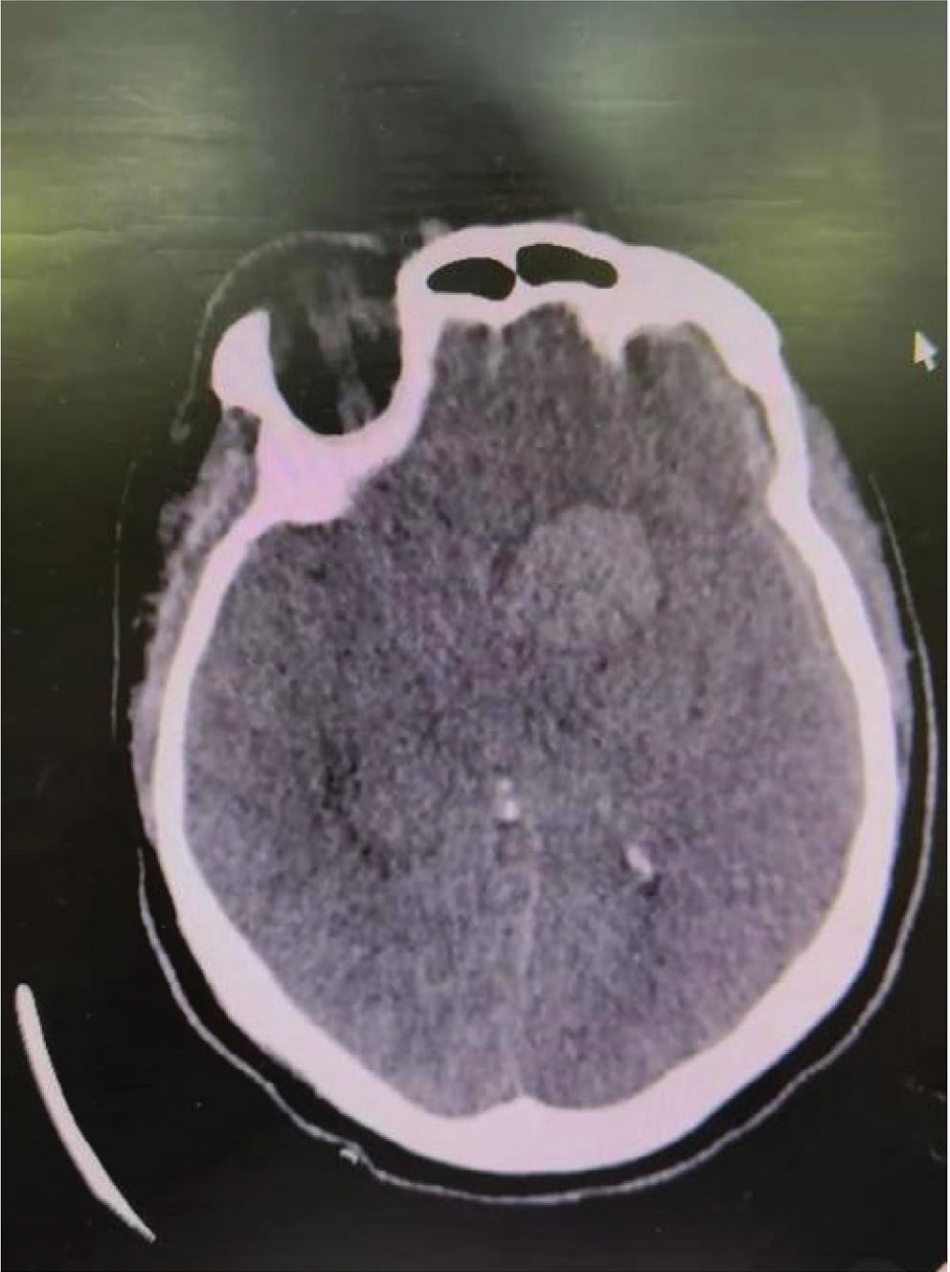
Patient's brain CT scan that showed a subarachnoid hemorrhage (SAH) with a left subdural hematoma.

On the third postoperative day, the patient unexpectedly developed a generalized tonic‐clonic seizure. A brain CT angiography was performed to investigate potential thromboembolic events, which showed a patent stent with no signs of new aneurysm formation (Figure 1). However, within 24 h, the patient experienced acute neurological deterioration characterized by decreased consciousness. Emergent CT imaging revealed a subarachnoid hemorrhage (SAH) along with a left‐sided subdural hematoma, indicating a significant complication (Figure 2).

**FIGURE 3:**
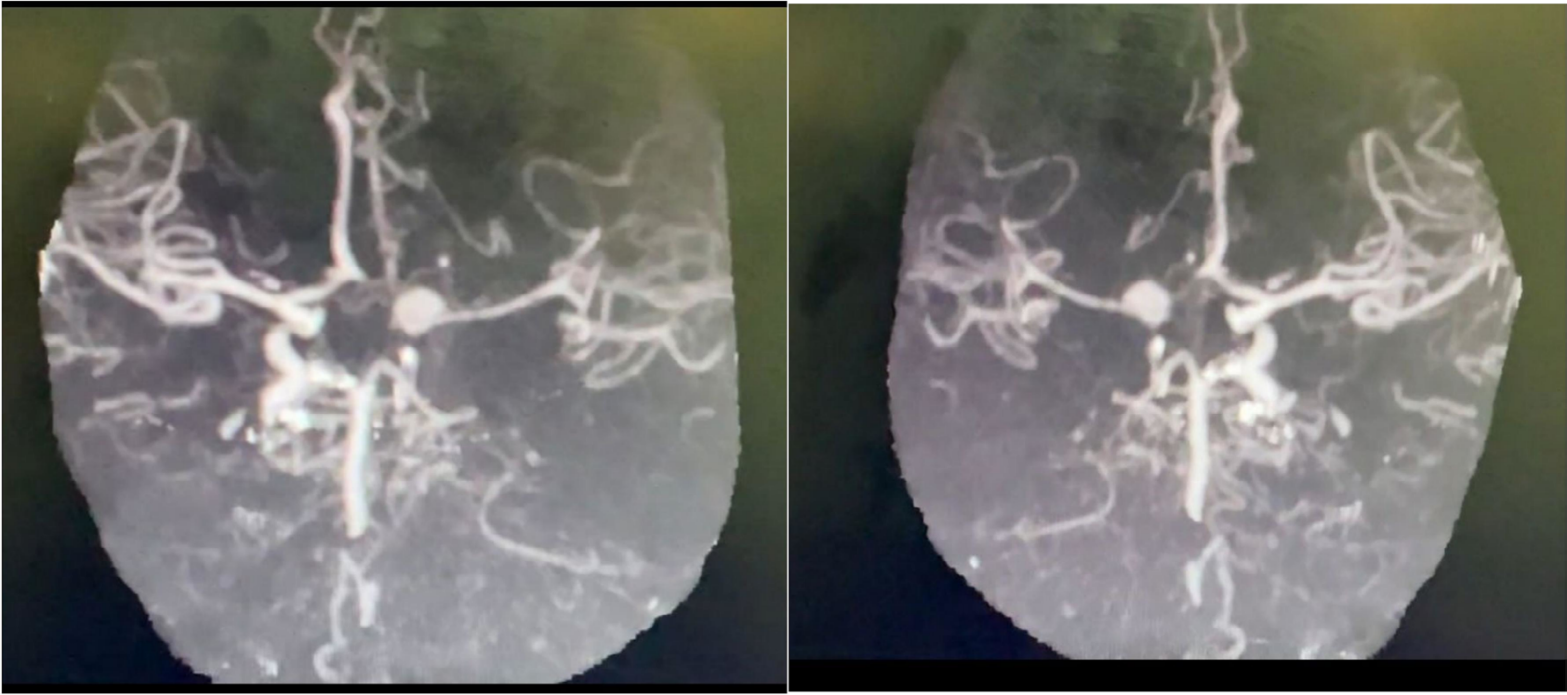
Dissecting pseudoaneurysm at the ICA bifurcation.

**FIGURE 4:**
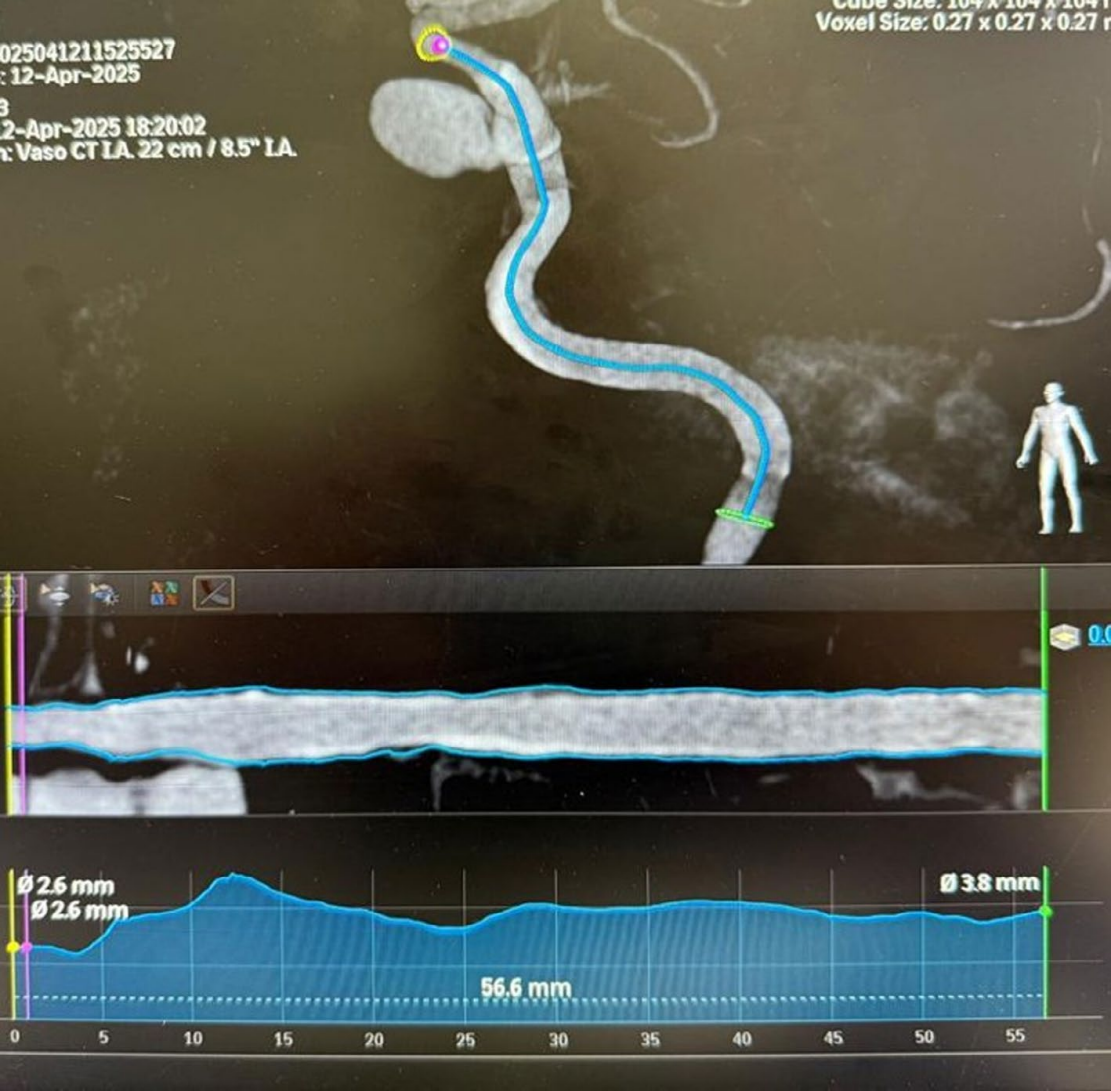
Analysis of the vascular wall demonstrated an intact arterial wall.

**FIGURE 5:**
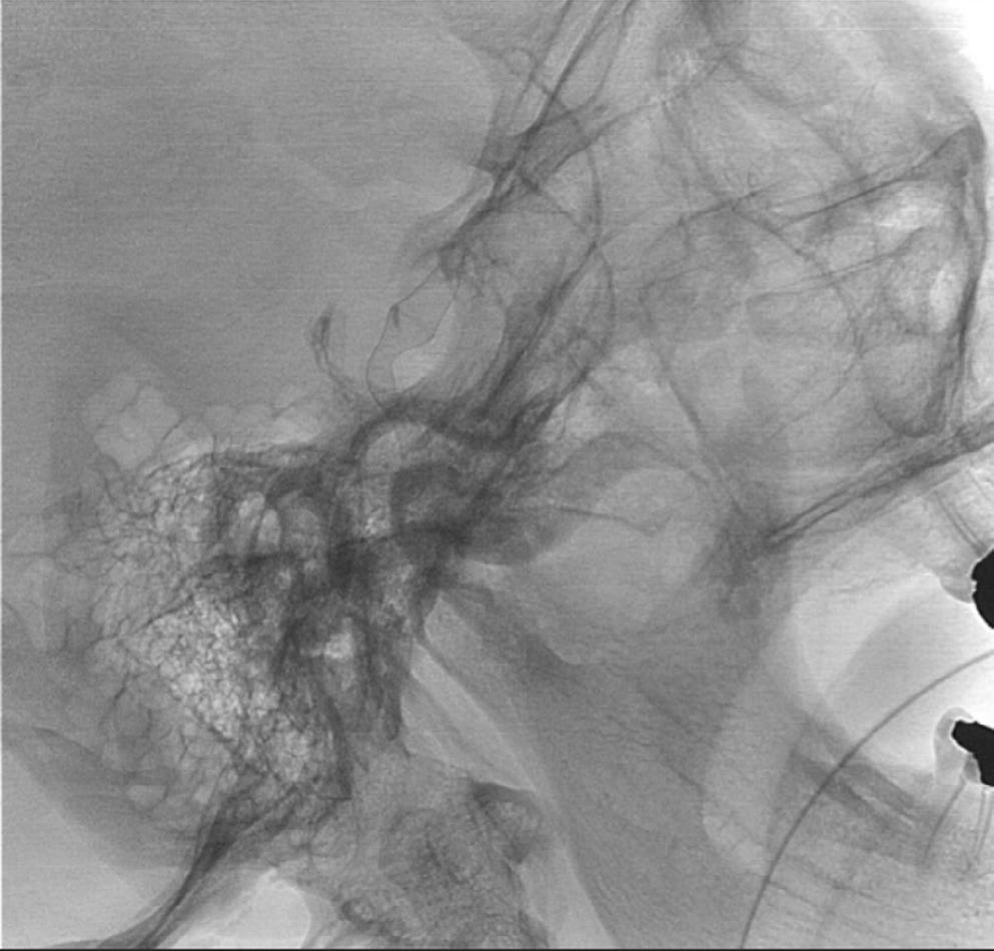
High‐resolution reconstructions demonstrating the implanted stent.

Repeat CT angiography (CTA) was performed, which identified a new dissecting pseudoaneurysm at the ICA bifurcation that had not been present in earlier imaging (Figure 3). Analysis of the vascular wall demonstrated an intact arterial wall with no signs of injury (Figure 4). Single‐shot appearance demonstrates well wall position of flow diverter (Figure 5).

The emergence of this new vascular lesion raised concerns regarding the stability of the patient's condition and the effectiveness of the previous interventions.

Despite aggressive medical management, including the optimization of blood pressure and seizure control, the patient's clinical condition continued to worsen. Ultimately, she expired, highlighting the severe complications that can arise in the context of vascular injuries and the challenges associated with managing such complex cases.

## 4 Discussion

Although rare, DIPH following flow diversion is a recognized phenomenon with high morbidity and mortality. The Windkessel hypothesis has been cited as a potential mechanism, suggesting that the loss of compliance from the excluded aneurysmal sac increases distal arterial pulsatility [6, 7]. However, emerging evidence suggests that this hypothesis may be insufficient to explain all cases, particularly those occurring very early after intervention.

Chien et al. developed an electrical analog to simulate post‐FD hemodynamic changes and found that flow diverters were unlikely to significantly alter distal pulse pressure, with estimated increases of only a few millimeters of mercury [6]. Their model and analysis suggest that Windkessel‐related changes alone cannot account for delayed hemorrhages. Importantly, their analysis was grounded in fluid dynamics and modeling, indicating that the physiological impact of aneurysm exclusion on downstream vessels may be minimal in terms of pressure amplitude.

These findings suggest that additional mechanisms beyond the Windkessel effect may be operative. In addition to hemodynamic alterations, delayed aneurysm rupture following flow‐diverter treatment has been associated with rapid intra‐aneurysmal thrombosis and subsequent inflammatory degradation of the aneurysm wall. Flow stagnation promotes thrombus organization, which may trigger proteolytic and inflammatory cascades that weaken the aneurysm wall, particularly in large and giant aneurysms [8, 9]. Perianeurysmal inflammatory changes have also been described after flow diversion and may reflect biologically active thrombus remodeling. Although our patient had a pseudoaneurysm rather than a typical saccular giant aneurysm, these observations further support the concept that hemorrhagic complications after flow diversion are multifactorial and cannot be explained solely by alterations in distal arterial compliance. The role of local vascular trauma (in this case, intraoperative carotid injury), endothelial dysfunction, acute vessel wall remodeling, thrombus‐related inflammatory responses, and altered vascular healing should all be considered. These mechanisms may act synergistically with hemodynamic alterations following flow‐diverter placement rather than representing mutually exclusive explanations [10, 11]. The role of local vascular trauma (in this case, intraoperative carotid injury), endothelial dysfunction, and acute vessel wall remodeling must be considered. In particular, injury‐induced inflammatory responses may accelerate dissection, pseudoaneurysm formation, and subsequent rupture. Moreover, the use of dual antiplatelet therapy, while essential to prevent stent thrombosis, may exacerbate the risk of hemorrhagic complications in patients with vulnerable vasculature.

Other proposed mechanisms for DIPH include disruption of the blood–brain barrier (BBB) due to increased shear stress or altered flow profiles, microembolization from thrombus debris, and regional perfusion changes. In this context, altered autoregulatory capacity may render distal brain regions more susceptible to sudden shifts in hemodynamics, especially when the ICA is manipulated or compromised. The lack of thrombus formation or stent occlusion in our case highlights the need to consider these alternative pathways.

Recent animal studies and computational fluid dynamic (CFD) modeling are beginning to support the multifactorial nature of post‐FD hemorrhage. Patient‐specific hemodynamic simulations may offer better risk stratification, particularly in those with prior vascular injuries, giant aneurysms, or complex anatomical configurations.

Platelet function testing was not performed in this patient. Consequently, the contribution of individual antiplatelet responsiveness, including possible excessive P2Y12 inhibition, cannot be assessed and should be considered a limitation of this report.

Ultimately, this case contributes to the evolving understanding of DIPH and underscores the need for tailored post‐FD monitoring strategies. Clinicians must maintain high vigilance for early post‐procedural neurological changes and consider advanced imaging and monitoring even in the absence of radiographic thrombotic events. As a single‐case report, causal relationships between flow diversion and the subsequent hemorrhagic complication cannot be established. Furthermore, the absence of platelet function testing limits the assessment of individual variability in antiplatelet response that contributed to the clinical course.

## 5 Conclusion

This case highlights a fulminant and early‐onset DIPH following flow diversion in a patient with prior carotid artery manipulation. The findings challenge the adequacy of the Windkessel hypothesis as a sole explanation and underscore the need to consider alternative mechanisms such as vessel wall fragility, hemodynamic shifts, and procedural factors. Clinicians must maintain high vigilance for early post‐procedural deterioration, particularly in high‐risk individuals. Future research involving patient‐specific modeling and prospective data collection is essential for improving risk stratification and preventing such devastating outcomes.

## Author Contributions

**Mohammad Samadian:** supervision, writing – review and editing. **Farid Kazemi:** conceptualization, investigation, writing – original draft, supervision, methodology, validation, visualization. **Mohammadreza Moznebiisfahani:** writing – review and editing, writing – original draft, conceptualization, investigation, methodology, supervision, validation, visualization, project administration. **Sajjad Khanbabazadeh:** data curation, supervision, conceptualization, investigation. **Mohammad Hossein Mirbolouk:** writing – review and editing, writing – original draft, conceptualization. **Feyz Ebrahimnia:** conceptualization, writing – original draft, methodology, supervision. **Seyad Tahar Mosavyan:** conceptualization, methodology, validation, visualization, writing – review and editing.

## Funding

The authors have nothing to report.

## Consent

Written informed consent was obtained from the patient to publish this report in accordance with the journal's patient consent policy.

## Conflicts of Interest

The authors declare no conflicts of interest.

## Data Availability

The data that support the findings of this study are available from the corresponding author upon reasonable request.
